# Commencements

**Published:** 1895-08

**Authors:** 


### COMMENCEMENTS.

### Western Dental College of Kansas City.

The fifth annual commencement exercises of the Western Dental College of Kansas City were held at the Auditorium, Kansas City, Mo., on Tuesday evening, March 5, 1895.

The annual address was delivered by Hon. Webster Davis, the faculty address by Dr. A. M. Wilson, and the valedictory by F. G. Worthly, D.D.S.

The number of matriculates for the session was one hundred and sixty-three.

The degree of D.D.S. was conferred on the following graduates by the dean, Dr. D. J. McMillen:

| NAME. | RESIDENCE. | NAME. | RESIDENCE. |
|---|---|---|---|
| Mrs. A. Baum | Kansas | R. W. Hood | Nebraska |
| L. A. Biggs | Missouri | Wm. A. Hazlett | Missouri |
| W. D. Bonfils | Missouri | Isaac Lesher | Kansas |
| Oscar Bruce | Kansas | J. F. McClure | Missouri |
| R. M. Burgess | Missouri | R. D. McIntosh | Missouri |
| Charles W. Corel | Missouri | Tom D. McGowen | Missouri |
| J. E. Chastain | Kansas | L. M. Moore | Kansas |
| E. W. Clark | Missouri | E. E. McCarty | Kansas |
| Alfred Daughaday | Missouri | R. L. Ready | Missouri |
| B. D. Gamble | Kansas | T. A. Sims | Indian Ty |
| A. Gihl | Missouri | C. C. Thomas | Missouri |
| E. H. Henry | Kansas | W. J. Tinker | Missouri |
| T. H. Howell | Indian Ty | B. L. Thorp | Missouri |
| G. A. Hartley | Kansas | F. G. Worthly | Missouri |
| W. N. Hall | Missouri | Irving Woolsey | Kansas |

Total, 30.

### University of Maryland—Dental Department.

The annual commencement exercises of the Dental Department of the University of Maryland were held at the Academy of Music, Baltimore, Md., on Friday afternoon, March 29, 1895, at 3 o'clock.

The annual address was delivered by Rev. Dr. C. Herbert Richardson, and the class oration by D. Edward Hoag, D.D.S.

The number of matriculates for the session was one hundred and eighty.

The degree of D.D.S. was conferred on the following graduates by Hon. John P. Poe, attorney-general of Maryland:

| NAME. | RESIDENCE. | NAME. | RESIDENCE. |
|---|---|---|---|
| W. C. Arthur | Pennsylvania | G. H. Lyndon | Ohio |
| W. B. Ashbrook | Pennsylvania | A. N. McKeever | West Virginia |
| H. C. Baker | West Virginia | G. C. Mann | Virginia |
| L. O. Bartlett | Cuba | C. F. Matthews | California |
| N. G. Bowbeer | Canada | E. H. Markley | Pennsylvania |
| L. D. Blackwelder | Pennsylvania | F. L. Parker | South Carolina |
| A. L. Bumgarner | Pennsylvania | R. W. Palmer | Virginia |
| W. J. Carter | Georgia | D. H. R. Patton | Nebraska |
| W. W. Cavers | New York | J. A. Rawlinson | Brazil, S. A |
| B. F. Copp | Colorado | S. C. Reynolds | Canada |
| J. C. Criswell | Pennsylvania | B. F. H. Rouse | Maryland |
| J. M. Fleming, Jr | North Carolina | L. St. C. Saunders | Nova Scotia |
| J. S. Geiser | Pennsylvania | D. Smithers | Maryland |
| T. O. Heatwole | Virginia | G. E. Starr | Maryland |
| H. C. Henderson | North Carolina | J. A. Stults | New Jersey |
| D. E. Hoag | New York | E. A. Tigner | Georgia |
| J. S. Hegner | Switzerland | G. S. Tigner | Georgia |
| J. H. Ihrie | North Carolina | N. McK. Wilson | Maryland |
| S. J. Irvine | Texas | C. A. Ward | Ohio |
| G. F. Jernigan | Texas | G. N. Ward | New York |
| B. Kritchevsky | Russia | M. E. Williams | New York |
| W. H. Kuich | Pennsylvania | S. H. Wolf | Pennsylvania |
| C. W. Link | West Virginia | | |

Total, 45.

### Birmingham Dental College.

The second annual commencement exercises of the Birmingham Dental College were held in Seal's Academy of Music, Birmingham, Ala., Thursday evening, March 28. 1895.

The address to the class was delivered by Captain Joseph F. Johnson, president of the board of trustees.

The number of matriculates for the session was twenty-four.

The degree of D.D.S. was conferred upon E. A. Wilson, of Alabama, by T. M. Allen, D.D.S., dean.

Total, 1.

### Western Reserve University—Dental Department.

The annual commencement exercises of the Dental Department of the Western Reserve University were held at Association Hall, Cleveland, Ohio, on Tuesday, March 5, 1895.

The annual address was delivered by Charles F. Thwing, D.D., president of the faculty.

The number of matriculates for the session was fifty-three.

The degree of D.D.S. was conferred on the following graduates:

| NAME. | RESIDENCE. |
|---|---|
| Frank H. Acker | Ohio |
| Leonard L. Bleasdale | Ohio |
| Allyn P. Buchtel | Ohio |
| Jay H. Burrows | Ohio |
| John W. Glas | Pennsylvania |
| Geo. A. Kennedy | Indiana |
| Dudley E. Mollen | Ohio |
| Percy O. Parsons | Ohio |
| James J. Rosensteel | Ohio |
| William W. Sherman | Ohio |
| Franklin J. Spargur | New York |
| David R. Stevenson | Ohio |
| Richard A. Suhr | Ohio |
| Louis S. Vinez | Ohio |
| Robert D. Wallace | Ohio |
| George N. Wasser | Pennsylvania |
| Henry J. Zoeckler | Ohio |

Total, 17.

### Royal College of Dental Surgeons of Ontario.

For the session of 1894–95 the students in attendance were: Freshmen, 50; juniors, 40; seniors, 45; total, 135. The following passed the senior examination and were admitted Licentiates of Dental Surgery:

J. C. Bansley.

W. C. Brown.

W. J. Bruce.

H. F. Burgess.

C. Bowerman.

W. Bell.

W. J. Brownlee.

C. W. Corrigan.

W. B. Cavanagh.

I. P. Cunningham.

L. H. Dawson.

George Emmett.

E. W. Falconer.

E. Fitzpatrick.

J. A. Fleming.

William C. Gowan.

William F. Ganton.

Richard Graham.

W. T. Griffin.

William S. Hall.

A. Irwin.

Fred. W. Ivory.

William C. Kennedy.

C. B. Lillie.

R. A. Marquis.

W. H. Mosley.

A. E. Mullin.

R. G. McLean.

T. E. Oliver.

E. W. Oliver.

K. Peaker.

J. P. Raleigh.

J. F. Ross.

H. C. Skinner.

N. Schnarr.

W. W. Thornton.

J. N. Wood.

A. J. Wyckoff.

F. Walters.

H. Wightman.

R. A. Willmott.

Total, 41.

All of the province of Ontario, Canada.

No formal commencement is held.

### Vanderbilt University—Dental Department.

The sixteenth annual commencement exercises of the Dental Department of Vanderbilt University were held in the chapel of the University, Nashville, Tennessee, on Wednesday, February 27, 1895.

The charge to the class was delivered by W. H. Morgan, dean, and the valedictory on the part of the class by H. S. Peach, B.A., D.D.S., of Alabama.

The number of matriculates for the session was one hundred and thirty-six.

The degree of D.D.S. was conferred on the following graduates by J. H. Kirkland, Chancellor of the University:

| NAME. | RESIDENCE. | NAME. | RESIDENCE. |
|---|---|---|---|
| J. U. Ball | Louisiana | J. M. Jacobs | Georgia |
| C. S. Bingham | Mississippi | L. H. Jeffries | Mississippi |
| J. S. Barter | Illinois | Y. Jones | Alabama |
| W. D. Beckwith | Georgia | W. T. King | South Carolina |
| A. D. Cage | Tennessee | P. A. Lee | Louisiana |
| M. A. Carroll | Alabama | J. J. Middleton | Louisiana |
| F. G. Campbell | Tennessee | G. C. McKennon | Arkansas |
| D. S. Dudley | Mississippi | W. Z. McElroy | Tennessee |
| C. P. Davis | Georgia | J. A. Naftel | Mississippi |
| C. M. Eddy | Illinois | P. O. Patureau | Louisiana |
| T. P. Fancheux | Louisiana | H. S. Peach | Alabama |
| J. A. Gholson | Tennessee | J. R. Pirtle | Kentucky |
| B. F. Gilmer | Illinois | J. L. Reeves | South Carolina |
| F. R. Greene | Florida | N. T. Rowland | Arkansas |
| L. T. Govee | Texas | W. J. Saunders, M.D. | Tennessee |
| J. G. Glass | Alabama | Lillie Selph | Tennessee |
| W. S. Hanner | Tennessee | W. H. Spurm | Louisiana |
| S. H. Hatcher | California | T. L. Whitehead | Virginia |
| J. R. Hiperger | Illinois | C. C. Winfrey | Kentucky |

Total, 38.

The next session beginning the first of October, will be extended to six months.

### Meharry Medical College—Dental Department.

The ninth annual commencement exercises of the Dental Department of Meharry Medical College (Central Tennessee College), were held at the Gospel Tabernacle, Nashville, Tenn., on Tuesday, February 5, 1895, in connection with those of the medical and pharmaceutical departments.

The address to the graduating classes was delivered by Rev. B. F. Rawlins, D.D., of Cincinncti.

The number of matriculates for the session was ten.

The degree of D.D.S. was conferred on the following graduates of the dental class by Rev. John Braden, D.D., president of the college: J. Abner Agnew, of West Virginia, and B. Frederic Barlow, of Texas.

Total, 2.

### University of Iowa—Dental Department.

The thirteenth annual commencement exercises of the Dental Department of the State University of Iowa were held at the Opera House, Iowa City, Iowa, on Monday evening, March 11, 1895.

The annual address was delivered by Professor C. S. Chase, A.M., M.D., of Waterloo.

The number of matriculates for the session was one hundred and fifty.

The degree of D.D.S. was conferred on the following graduates by Charles A. Schaeffer, Ph.D., president of the university:

| NAME. | RESIDENCE. | NAME. | RESIDENCE. |
|---|---|---|---|
| J. C. Alexander | Wisconsin | L. G. Lawyer | Iowa |
| Mrs. W. J. Addenbrooke | Wisconsin | I. S. Mahan | Iowa |
| W. J. Addenbrooke | Wisconsin | M. B. McCabe | Iowa |
| Grant Bruner | Iowa | Martha S. McCoy | Kansas |
| C. C. Colby | Nebraska | J. A. McErlain | Iowa |
| H. M. Eaton | Iowa | C. A. Palmer | Iowa |
| G. H. Edginton | Iowa | A. O. Petersen | Illinois |
| R. C. Grimm | Iowa | E. A. Phillips | Iowa |
| F. E. Hart | Nebraska | F. J. Ruggles | Iowa |
| F. B. Hyatt | Iowa | J. I. Tomy | Iowa |
| H. R. Hitchins | Iowa | C. M. Work | Iowa |
| P. H. Jones | Iowa | B. E. Wright | South Dakota |
| W. L. Lamb | Wisconsin | | |

Total, 25.

### Columbian Dental College.

The third annual commencement exercises of the Columbian Dental College were held at Kimball Hall, No. 243 Wabash Avenue, Chicago, Ill., on Saturday, March 9, 1895, at 2 P. M.

The opening address was delivered by S. Allen Wilson, B.S., D.D.S.; the class valedictory by R. Fred. Throckmorton, M.D., D.D.S., and the closing address by Edward C. Nichols, LL.B.

The number of matriculates for the session was forty-eight.

The degree of D.D.S. was conferred on the following graduates by J. D. Shugart, D.D.S., dean:

| NAME. | RESIDENCE. | NAME. | RESIDENCE. |
|---|---|---|---|
| S. G. Abbott | Wisconsin | A. P. Lusk | Wisconsin |
| D. A. J. Ferner | Illinois | W. Fred. Nickel | Illinois |
| W. Herbert Fisher | Canada | C. H. Parker | Illinois |
| J. E. Kraft | Missouri | R. Fred. Throckmorton | Iowa |
| W. E. Little | Illinois | Albert F. Wineman | Illinois |

Total, 10.

### Medical Department of the University of Denver.

The fourteenth annuul commencement exercises of the Medical Department of the University of Denver were held Tuesday evening, April 16, 1895.

The address to the graduates in Dentistry was delivered by the Hon. B. M. Malone.

The degree of D.D.S. was conferred on the following graduates by Chancellor McDowell: William Curtis Ball and Louis Arthur Whitney.

Total, 2.

### Ohio Medical University—Dental Department.

The third annual commencement exercises of the Ohio Medical University, including the Dental Department, were held in the Board of Trade Auditorium, Columbus, Ohio, on Tuesday evening, March 19, 1895.

The annual address was delivered by S. McChesney, D.D., and the address on behalf of the faculty by G. M. Waters, B.A., M.D.

The number of dental matriculates for the session was fifty-four.

The degree of D.D.S. was conferred on the following graduates by J. M. Dunham, A.M., M.D, president of the board of trustees:

Enoch Francis Downing.

George Howard Henry.

Elmer Earnest Latham.

Arthur Nettleton Lindsey.

Dr. T. Gray Merrett.

Charles Stewart Millen.

George Prince Stephenson.

Halstead Robert Wright.

Total, 8.

### National University—Dental Department.

The annual commencement exercises of the Dental Department of the National University were held (in connection with those of the Medical Department) in Metzerott Music Hall, Washington, D. C., on Tuesday evening, May 14, 1895.

The address to the graduating classes was delivered by Professor William Mercer Sprigg, M.D., and the valedictory address by Harry Jerome Allen, D.D.S., M.D.

The number of matriculates for the session was thirty-six.

The degree of D.D.S. was conferred on the following graduates of the dental class by the chancellor of the university:

| NAME. | RESIDENCE. | NAME. | RESIDENCE. |
|---|---|---|---|
| Harry J. Allen | Dist. Columbia. | Albert E. McConnell | Dist. Columbia |
| Llewellyn F. Davis | Maine | Ambler A. Marsteller | Virginia |
| Harry P. Davis | Virginia | William D. Monroe | Virginia |
| William W. Hodges | Pennsylvania | Nora Moyer | Pennsylvania |
| Frank D. Magnus | Connecticut | | |

Total, 9.

### Columbian University—Dental Department.

The eighth annual commencement of the Dental Department of the Columbian University was held at Metzerott Music Hall, Washington, D. C., on Tuesday. April 30, 1895, at 8 o'clock P. M.

The address to the graduates was delivered by Professor H. B. Noble, D.D.S., and the valedictory by Henry Knowles, D.D.S.

The number of matriculates for the session was sixty-four.

The degree of D.D.S. was conferred on the following graduates by the President S. C. Green, D.D.

| NAME. | RESIDENCE. | NAME. | RESIDENCE. |
|---|---|---|---|
| Don F. Aguilera | U. S. Colombia | Eugene L. LeMerle | Dist. Columbia |
| Joseph Cohen | Texas | Charles W. Orr | New York |
| James W. Davis | Maine | Will K. Petty | Dist. Columbia |
| William S. Gustin | Ohio | Harry D. Parsons | New York |
| William Hislop | Ontario | William H. Trail | Maryland |
| Robt. E. L. Hackney | Virginia | Ernest C. Varela | California |
| J. Everett Keene | Dist. Columbia | Israel G. Warfield | Maryland |
| Henry Knowles | Georgia | | |

Total, 15.

### Cleveland University of Medicine and Surgery—Dental Department.

The annual commencement exercises of the Dental Department of the Cleveland University of Medicine and Surgery were held (in connection with those of the medical department) at Association Hall, Cleveland, Ohio, on Tuesday evening, March 26, 1895.

The number of matriculates for the session in the dental department was twenty-seven.

The degree of D.D.S. was conferred on the following graduates:

| NAME. | RESIDENCE. | NAME. | RESIDENCE. |
|---|---|---|---|
| Samuel Howard Stevens | Ohio | Edwin Stanton Kiplinger | Ohio |
| Louis Amedius Keller | Ohio | James Milford Chambers | Ohio |
| Jay Thorne Newton | Ohio | | |

Total, 5.

### The Boston Dental College.

The twenty-eighth annual commencement exercises were held in Berkley Temple, Boston, June 20th, at 8:00 P. M.

The address was delivered by Prof. Geo. A. Bates, D.D.S., and the valedictory by Dr. A. W. Noll, D.D.S. The graduating class, numbering forty-two, received their degrees from the hands of the President of the College, Dr. I. J. Wetheree, D.D.S., the names of which are as follows:

John Francis Ager.

Arthur David Allard.

Walter Ira Boynton.

Chauncy Morris Carpenter.

William Martin Flynn.

Helen Mar Henry.

Arthur Horsfall.

Ernest Hosmer.

Roscoe Henry Hull.

Walter Mayo Kelty.

John William Kenney.

John Walter Keyes.

Charles Bingley Little.

Carrie Gertrude Locke.

Guy Bernard Manzer.

George Henry Martin.

John Amon Dexter Mills.

James Thomas Moriarty.

James Edwin Nealis.

Albert William Noll.

Daniel Butler Nye.

John William Pomfret.

Percy Morris Porter.

James Francis Potter.

Jeremiah Patrick Reardon.

John Lawrence Reilly.

William Thomas Reilly.

Francois Xavier Medard Polycarpe Renaud.

Walter Edwin Rowe.

William Alexander Rowe.

George Atherton Sleeper.

Westford Marlow Taylor.

George Olivir Tessier.

Edmond Emanuel Vadnais.

Charles Everett Walker.

Richard Ambrose Walsh.

Lewis Lincoln Warren.

Oscar Emery Wasgatt.

Conrad Ogilvie Hall Webster.

Roscoe Livingston Wentworth.

Elmer Lorendo White.

George William Yale.

Total, 42.

### University of Michigan—Dental Department.

The twentieth annual commencement of the Dental Department was held in connection with the general commencement of the University, in University Hall, June 27, at 10 o'clock A. M.

The commencement address was delivered by James H. Canfield, LL.D., President of the Ohio University.

There were enroled in this department one hundred and eighty-seven, and in the graduating class forty-seven.

The degree of D.D.S. was conferred by Dr. J. B. Angell, President of the University, upon the following named persons:

Douglas Anderson.

Archibald Elmer Ball.

Amos Barnes.

Orville M. Barton.

Alfred Lee Beatie.

Joseph Henry Billmeyer, Jr.

Joseph Augustin Bucknall.

Fred. Crittenden Clapp.

Lewis Emmett Coonradt.

Mary Bruyn Crans, B.S. University of North Dakota.

Fred. Ellsworth Dodge.

John B. Dowdigan.

Edmund Dubuis, D.E.D.G., Dental Dep't, Univ. of Geneva, Switzerland

Walter Gideon Dunham.

George Frederick Fiddyment.

Van Camp Carratt, D.D.S., American College of Dental Surgery.

Fred. Pratt Graves.

Arche Greenwood Hicks.

Harry Benson Hinman.

Marshal Luther Howver.

Arthur Stimson Kennedy.

John Fredrik Henry Kuyper.

Walter Allen Lapman.

Harry Hallenbeck Lauderdale.

Albert Leland LeGros.

William Gustave Lentz.

Frank Eugene McLaughlin.

Joseph Merckens.

Daniel Merner.

John Henry Neeley.

George A. Parmenter.

Clarence Fletcher Piper.

Harry Benedict Respinger, D.E.D.G., Dental Department, University of Geneva, Switzerland.

Burt Townsend Ruthruff.

Francis Frederick Scott.

Newton J. Smith, Jr.

Charles Bradford McCall Southwick.

Joseph Herman Stromier.

Clifford Paul Sweny.

Andrew Roane Thorpe, A.B., St. Vincent's Coll.

Christian Leonard Theurer.

George McAlpine Tyng.

Perley Tapler Van Ornum.

Elizabeth von Bremen.

Friedrich von Widekind.

DOCTOR OF DENTAL SCIENCE.

Will Hamilton Van Deman, D.D.S.
Charles Traver Whinery, D.D.S.

Total, 47.

### Kansas City Dental College.

The thirteenth annual commencement exercises of the Kansas City Dental College were held at the Auditorium, Kansas City, Mo., on Friday evening, March 1, 1895.

The salutatory was delivered by William A. DeBerry, D.D.S.; the faculty address by Professor Rathbone; the valedictory by Amos L. Curry, D.D.S., and the annual address by Bishop Hendricks.

The number of matriculates for the session was one hundred and fifty-three.

The degree of D.D.S. was conferred on the following graduates by Dr. L. C. Wasson:

| NAME. | RESIDENCE. | NAME. | RESIDENCE. |
|---|---|---|---|
| George A. Adcock | Missouri | Joseph Lutz | Kansas |
| George R. Burns | Indiana | Jesse Miller | Missouri |
| John Barber | Missouri | Samuel M. McDonald | Missouri |
| Ferdinand J. K. Bush | California | Milton B. Metzler | Missouri |
| Walter E. Blackwell | Missouri | Franklin K. Munday | California |
| William H. Brosman | Nebraska | James A. Nevitt | Missouri |
| Theron I. Branstetter | Missouri | Thomas G. Newell | Minnesota |
| Julian C. Bogue | Oregon | Charles C. Needham | Missouri |
| Harry W. Balthrope | Mississippi | Charles M. Palmer | Colorado |
| Edward L. Conrad | Missouri | Abram P. Preston | California |
| Amos L. Curry | California | Harry L. Prevost | Missouri |
| Henry W. Cussons | Nebraska | Frederick H. Riley | Missouri |
| John W. K. DeHaven | Missouri | Overton M. Saul | California |
| William A. DeBerry | Missouri | Charles N. Smith | Kansas |
| Leland P. Davis | Nebraska | Albert R. Streicher | Missouri |
| William E. Furrow | Oklahoma | Arch. Thompson | Missouri |
| Charles I. Glenn | Missouri | John W. Vaughn | Kansas |
| Walter R. Gwin | California | Walter Z. Wright | Kansas |
| George J. Geenen | Illinois | Eric C. Watkins | Kansas |
| Joseph T. Longstreth | Missouri | | |

Total, 39.

### University of Tennessee—Dental Department.

The annual commencement exercises of the Dental Department of the University of Tennessee were held at Nashville, on Thursday, March 21, 1895.

The charge to the dental graduates was delivered by Professor Joseph P. Gray, M.D., D.D.S., and the valedictory by W. A. Titus, D.D.S.

The number of matriculates for the session was fifty-eight.

The degree of D.D.S. was conferred on the following graduates by Thomas W. Jordan, A.M., acting president of the university:

| NAME. | RESIDENCE. | NAME. | RESIDENCE. |
|---|---|---|---|
| J. W. Blair | Tennessee | J. R. Pendleton | Missouri |
| S. A. Garth | Missouri | J. I. Pettus | Arkansas |
| F. M. Gayle | Louisiana | J. T. Phillips | Alabama |
| E. D. Hancock | Kentucky | N. A. Royer | Tennessee |
| Clarence Hudson | Missouri | H. R. Rouse | Indiana |
| J. B. Love | Tennessee | A. C. Seiser | Missouri |
| J. E. McGowan | Tennessee | W. A. Titus | Pennsylvania |
| L. H. New | Tennessee | | |

Total, 15.

